# Ethical Pre-Conceptions and the Physicians’ Duty

**Published:** 1917-10

**Authors:** 


### Ethical Pre-Conceptions and the Physicians' Duty.

This article is addressed especially to young practitioners, because of a recollection of an early state of bewilderment in adjusting different ethical codes.

ALCOHOL. You may have been brought up to regard this agent as not only injurious but as essentially and peculiarly iniquitous. Without entering into therapeutic disputes, or discussing the indications and contra-indications of alcohol as a drug, you should, as a physician, regard it exactly as you would strychnine, sodium bromid or any other therapeutic agent. A person accustomed to taking alcoholic beverages, especially strong spirits of any kind, and more especially if he is accustomed to taking them regularly and without becoming intoxicated in the ordinary sense of the word, is very likely to have an attack of delirium tremens precipitated by any shock, even that of an acute medical ailment. Under such circumstances, the continuance of the alcohol, in moderate dose, is a matter of prophylactic common sense.

TOBACCO. In many cases, severe nervous symptoms complicate surgical and medical cases, from the withdrawal of tobacco suddenly. The patient may be so imbued with the inappropriateness of tobacco to a confinement to bed for a serious trouble, that he may not even ask to be allowed to smoke or chew. In such cases, moderate indulgence often does no harm, on the contrary, it may avert nervous complications very much as moderate continuance of alcohol prevents delirium tremens. Do not forget that some women have the tobacco habit. Without advocating the use of tobacco, the medical adviser, in mild cases, should be guided by actual experience, general and individual and should not interdict its use simply as a reform measure. The physician should not yield to popular prejudice in regard to different forms of use of tobacco. Chewing and inhaling smoke obviously increase the absorption per unit of tobacco used and exaggerate the respective local effects. But it is unwise to attempt to reform a tobacco chewer for aesthetic reasons in an emergency. A package of cigarettes contains almost exactly the amount of tobacco contained in an average cigar and the flame is more exposed to oxygen. There is the same logic in advising a man against cigarettes and in favor of cigars or a pipe, as in advising him to drink large quantities of whisky instead of small amounts of beer.

SEXUAL AND OTHER IMMORALITIES. The physician, like the clergyman and teacher, is rightly expected to take a high moral ground, both in his personal life and in his influence. But, as an advisor, in a professional capacity, his first duty is his patient's welfare and he has no right to tell lies for their remote uplifting effect. To tell an inexperienced boy that masturbation is the principal cause of insanity, weighs heavily toward making it a cause in the particular case. To force an ill assorted marriage on a patient, for the sake of bringing about the conventional, respectable ending of an unfortunate amour, may result in untold misery. To attempt to secure moral living by exaggerated statements of venereal and other risks is not only wrong because it involves lying but it is, in the long run, futile. You can scare most inexperienced persons into believing anything for a while but most of them will discover the truth from subsequent observation and your influence will be gone. Do your duty as a physician, in all such cases and then add whatever admonition you may feel that you should, as a friend and moralist, but tell the truth.

THE RIGHTS OF THE WRONG KIND OF PEOPLE. You have very likely been brought up to regard prostitutes, kept women, fast men, drunkards, saloonkeepers, gamblers and criminals as highly undesirable. But remember, that when sick or injured, these persons have the same claim upon you as a physician, as the most reputable members of society. This does not mean merely that you will refrain from an attempt at improving general standards of social morality by killing them off when they come under your professional care. Nor does it mean merely that you will not accomplish the same end by neglect. It implies the same promptness in attendance, the same courtesy and the same consideration that you give to any other patient. You will perhaps be surprised to learn that the most nefarious occupations are not inconsistent with kindness of heart, industry and many other virtues, that the depths of indecency do not always efface an appreciation of delicacy of manner and speech. In any vocation, one has the right to aim for the most desirable patronage but, in medical practice, it is questionable whether one ever has the right to close the door to a patient simply because he or she is not respectable, clean and "desirable." The ethics of the profession, established custom and the law itself, all support the contention that the relation of patient and physician is one of confidence, even of secresy. You owe no detective service to the community from the opportunities of uncovering crime that arise in practice. On the other hand, you owe to no patient the commission of a crime under the guise of relieving suffering nor participation in a crime of other nature.

SOCIAL DISTINCTIONS. After the almost universal teaching of democratic principles in schools and in ordinary associations, the very frank recognition of social differences in the discussion of disease, is often a surprise to immature medical students. But, in medicine, facts rather than theories, are of paramount importance. One of the cruelest facts is that men run greater risk of disease and accident and greater risk of death when sick or disabled, because of poverty. On the other hand, one of the greatest merits of the medical profession is that, so far as it is itself concerned, wealth and social rank are as nearly as possible leveled when it comes to the actual combat against disease and death. But, in dealing with medical and surgical cases, democratic theories must often be set aside in favor of existing facts, in determining various details of the management.

---

Does it ever occur to the medical profession that advertisers deserve their special consideration? Quite aside from the fact that the great majority of advertisers in medical journals have something to sell of real merit, and that, like a man before a court of law, as plaintiff or defendant, every advertiser ought to have the right to a respectful consideration of his contention, whatever our preconceived ideas, the men who are advertising their goods and services are bearing the greater part of the tax for one of the greatest means of education. Every periodical depends upon advertisers rather than on subscribers, for its support and there is no other educational factor so important as periodical literature in all its manifold branches—except class room instruction. Let the man who tries to shirk his share of the burden and bother you with circulars, know that his stuff goes promptly to the W. P. B. We are making no selfish plea, we do not care whether a firm advertises in this particular journal or not, so long as he is doing his part among journals generally. We ask no discrimination. Simply discard all circulars, excepting catalogues, etc.

